# Hsa_circ_0137008 suppresses the malignant phenotype in colorectal cancer by acting as a microRNA-338-5p sponge

**DOI:** 10.1186/s12935-020-1150-1

**Published:** 2020-03-04

**Authors:** Zhanfeng Yang, Jingjing Zhang, Danghui Lu, Yan Sun, Xinyong Zhao, Xiaoqiong Wang, Wen Zhou, Qunli He, Zhi Jiang

**Affiliations:** 1 Department of Medicine, Zhengzhou University of Industry Technology, 16 Xueyuan Road, Xinzheng, 451100 Henan China; 2 Department of Vascular Surgery, Henan Provincial People’s Hospital, 7 Weiwu Road, Zhengzhou, 450000 Henan China; 3 The Second Affiliated Hospital of Guangzhou University of Chinese Medicine, 111 Dade Road, Guangzhou, 510120 China

**Keywords:** Colorectal cancer, Hsa_circ_0137008, microRNA-338-5p, Proliferation, Migration, Invasion

## Abstract

**Background:**

Circular RNAs (circRNAs) have been shown to play a crucial role in tumorigenesis. In this study, we investigated the function of hsa_circ_0137008 and its underlying molecular mechanism in colorectal cancer (CRC).

**Methods:**

Gene expression was conducted by quantitative real-time PCR or western blot. Functional experiments were performed by cell count kit-8, colony formation assay, wound healing, and transwell assays. Luciferase reporter assay and RNA pull-down assay were performed to investigate the molecular mechanism of hsa_circ_0137008 in CRC. In addition, the xenograft tumor model was applied to determine the role of hsa_circ_0137008 in vivo.

**Results:**

Downregulation of hsa_circ_0137008 was observed in CRC tissues and cell lines. Functionally, overexpression of hsa_circ_0137008 inhibited the proliferation of CRC cells, as indicated by the inhibition of proliferative protein expression (Ki67 and PCNA), reduced cell viability and colony formation ability. Upregulation of hsa_circ_0137008 suppressed the migration, invasion, and epithelial to mesenchymal transition (EMT) of CRC cells. Mechanically, hsa_circ_0137008 negatively regulated the expression of microRNA-338-5p (miR-338-5p). Furthermore, hsa_circ_0137008 abated the miR-338-5p mediated promotion on CRC cell progression. Tumor suppressive function of hsa_circ_0137008 was validated in vivo.

**Conclusion:**

These findings highlighted the fact that overexpression of hsa_circ_0137008 inhibited the progression of CRC via sponging miR-338-5p, suggesting that hsa_circ_0137008/miR-338-5p axis is a principal regulator of CRC tumorigenesis.

## Background

Colorectal cancer (CRC), a malignant tumor of the colon or rectum, is one of the main reasons of cancer-related mortality in the world [1–3]. Although in recent years, a considerable progress has been made in the diagnosis and therapy of CRC, the therapeutic efficacy of CRC patients is still poor [4, 5]. Thus, elucidation of the mechanism underlying CRC progression is of great significance to find a novel therapeutic strategy for CRC.

Circular RNAs (circRNAs) are a new subtype of endogenous non-coding RNAs, which is characterized by a covalently closed continuous loop [6–9]. CircRNAs have been proposed to participate in regulating a wide range of gene expression. Abnormal expression of circRNAs was reported to be linked to the development of human diseases, which make them potential biomarkers and possible therapeutic targets for human diseases [10]. Therefore, circRNA research has become the hotspot of the study of various human diseases. Previous studies have showed that circRNAs were closely related to the occurrence and development of human cancers, such as breast cancer [11], gastric cancer [12], and CRC [13]. hsa_circ_0137008, a newly identified circRNA, has been found to be reduced in CRC tissues [14]. However, we still lack a clear understanding of the functional role of hsa_circ_0137008 in CRC.

MicroRNAs (miRNAs), a class of small non-coding RNAs with approximately 20 nucleotides, able to directly bind to the 3ʹ untranslated region of mRNAs, thereby regulating mRNA degradation and translational inhibition [15, 16]. MiRNAs have been reported to be related to various physiological and pathological processes [17]. In recent years, an increasing number of studies suggests that miRNAs serve as the oncogenic or tumor-suppressive factors in the progression of human cancers [18]. MiR-338-5p is a member of the miR-338 family and has been documented to be involved in the development of multiple human cancers, such as melanoma [19], hepatocellular carcinoma [20], and gastric cancer [21]. MiR-338-5p has been reported to be differentially expressed in CRC tissues, which make it a classifier for CRC detection [22]. Previous researches have suggested that circRNAs acted as miRNA sponges to participate in the progression of human cancers [23]. However, whether hsa_circ_0137008 regulates CRC progression through sponging miR-338-5p remains largely elusive till now.

In the current study, we investigated the function and molecular mechanism of hsa_circ_0137008 in CRC cells. We found that hsa_circ_0137008 was significantly downregulated in CRC tissues and cell lines. Mechanistically, hsa_circ_0137008 acted as miR-338-5p sponge to regulate CRC progression, indicating that hsa_circ_0137008 may be a potential therapeutic strategy for CRC patients.

## Materials and methods

### Patient tissue samples

Human CRC tissues (*N* = 30) and adjacent normal tissue samples (*N* = 30) were collected from CRC patients at The Second Affiliated Hospital of Guangzhou University of Chinese Medicine. The patients had not received any treatment before surgery. After excision, the tissues were immediately plunged into liquid nitrogen and stored at − 80 °C. This study was approved by The Second Affiliated Hospital of Guangzhou University of Chinese Medicine and in accordance with the ethical standards formulated in the Helsinki Declaration, and written informed consents were obtained from all the participants.

### Cell culture

Human normal colon epithelial cell line (FHC) and CRC cell lines (HT29, HCT116, HCT8, LOVO, SW480, and SW620) were obtained from the American Tissue Culture Collection (ATCC, Manassas, VA, USA). The cells were cultured in Roswell Park Memorial Institute-1640 medium (RPMI-1640; HyClone, Logan, UT, USA) containing 10% fetal bovine serum (FBS; Gibco, Grand Island, NY, USA) at 37 °C in a humidified atmosphere with 5% (v/v) CO_2_.

### Cell transfection

Empty pcDNA3.1 vector (Vector) and hsa_circ_0137008-overexpressing plasmid (circ_0137008) were synthesized by GeneCopoecia (Guangzhou, China). While miR-338-5p mimic and miRNA negative control (miR-NC) were obtained from RiboBio (Shanghai, China). SW480 and HCT116 cells were transfected with these molecules by using Lipofectamine 2000 (Invitrogen, Carlsbad, CA, USA), according to the manufacturer’s instructions. At 48 h later, the cells were collected for the following experiments.

### CircRNA plasmid stable transfection

Transfection was performed using Lipofectamine 2000 reagent (Invitrogen) according to the manufacturer’s protocol. The transfected cells were selected by screening of G418 (Sigma-Aldrich, St Louis, MO, USA) for 6 weeks. Surviving cells were subjected to identification of overexpression efficiency. The cells stably overexpressing hsa_circ_0137008 could be used for further experiments.

### Quantitative real-time PCR (qRT-PCR) assay

Total RNA was isolated from tissues and cells using TRIzol reagent (Invitrogen) according to the manufacturer’s instructions. The quality of isolated RNA was determined using NanoDrop ND-1000 spectrophotometer. Then the RNA was reverse transcribed into cDNA using PrimeScript RT Reagent Kit (Takara, Dalian, China), following by qRT-PCR analysis on Bio-Rad CFX96 system (Bio-Rad, Hercules, CA, USA). TaqMan miRNA assay was performed to examine the expression of miR-338-5p. The relative expression levels of hsa_circ_0137008 and miR-338-5p were calculated by the 2^−ΔΔCt^ method, using β-actin and U6 as internal reference genes, respectively.

### Western blot

After transfection, SW480 and HCT116 cells were collected, followed by protein extraction using RIPA lysis buffer. After 10% sodium dodecyl sulphate–polyacrylamide gel electrophoresis, isolated proteins were electro-transferred to polyvinylidene fluoride membranes and then blocked for 60 min with 5% skim milk. Subsequently, the membranes were incubated with primary antibodies from Boster (Wuhan, China) at 4 °C overnight, followed by incubation with horseradish peroxidase-conjugated secondary goat antibody (catalog number: BA1056) from Boster for 1 h at room temperature. The primary antibodies used in this experiment were list as follows: mouse anti-Ki-67 antibody (catalog number: M00254-1, Boster), mouse anti-proliferating cell nuclear antigen antibody (PCNA, catalog number: BM0104, Boster), rabbit anti-β-actin antibody (catalog number: BM3873, Boster), rabbit anti-E-cadherin antibody (catalog number: M00063-3, Boster), rabbit anti-Vimentin antibody (catalog number: BM4029, Boster), mouse anti-N-cadherin antibody (catalog number: BM1573, Boster).

### Cell proliferation assay

The proliferation of SW480 and HCT116 cells was tested by using Cell Counting Kit-8 (CCK-8; Dojindo Molecular Laboratories, Japan). After transfection, SW480 and HCT116 cells were seeded in 96-well plates (1 × 10^4^ cells/well) and cultured for indicated times at 37 °C with 5% (v/v) CO_2_. Then, the cells were treated with 10 µl CCK-8 solution at 37 °C for 2 h. Subsequently, the absorbance of each well at 450 nm was assessed by mean of a spectrophotometer.

### Colony formation assay

Colony formation assay was used to evaluate the proliferation of SW480 and HCT116 cells. After transfection, SW480 and HCT116 cells were plated into 6-well plates. The cells were incubated at 37 °C in a humidified incubator with 5% (v/v) CO_2_ for 14 days. Afterward, the cells were fixed with glutaraldehyde and stained with crystal violet. Colonies were pictured and counted under a stereomicroscope. Image J software was used to calculate the number of colonies (more than 50 cells).

### Migration assay

The migration of SW480 and HCT116 cells was measured by wound-healing assay. After transfection, SW480 and HCT116 cells (1 × 10^5^ cells/well) were seeded in 6-well plates and incubated for 24 h. A straight scratch was introduced by dragging the tip of the sterile pipette across the monolayer of SW480 and HCT116 cells. After washing with sterilized phosphate buffer saline (PBS), the cells were incubated in serum-free medium. The width of wound was pictured at 0 h and 48 h under a microscope.

### Invasion assay

Transwell invasion assay was performed to evaluate the invasion of SW480 and HCT116 cells. After transfection, SW480 and HCT116 cells (5 × 10^4^ cells/well) were suspended in serum-free medium and plated into the upper chamber pre-coated with Matrigel (BD Biosciences, San Jose, CA, USA). The lower chamber was filled with RPMI-1640 medium plus 10% FBS. After 48 h of incubation, a cotton swab was used to remove the cells on the upper surface of the chamber. The invaded cells on the lower surface of the chamber were fixed with 4% paraformaldehyde, and then stained with 0.1% crystal violet. The stained cells were counted with an inverted microscope (Thermo Fisher Scientific, Waltham, MA, USA).

### Subcellular fraction assay

The nuclear and cytoplasmic RNA from SW480 and HCT116 cells were isolated using the PARIS Kit (Life Technologies, Carlsbad, CA, USA) following the manufacturer’s instructions, and then tested for hsa_circ_0137008 expression by qRT-PCR.

### Luciferase reporter assay

The online database CircInteractome (https://circinteractome.nia.nih.gov) was used to predict the potential miRNA downstream of hsa_circ_0137008. The wild type (WT) and mutated (MUT) sequence of hsa_circ_0137008 were cloned into pcDNA3.1 vector. Then, SW480 and HCT116 cells were plated into 24-well plates and co-transfected with circ_0137008-WT or circ_0137008-MUT and miR-NC or miR-338-5p using Lipofectamine 2000 (Invitrogen). At 48 h after transfection, dual luciferase reporter assay kit (Promega, Madison, WI, USA) was used to measure the luciferase activity, according to the manufacturer’s manual.

### RNA pull-down assay

SW480 and HCT116 cells were transfected with biotinylated miR-NC (Bio-NC) or biotinylated miR-338-WT (Bio-miR-338-WT) or biotinylated miR-338-MUT (Bio-miR-338-MUT), which were commercially synthesized by RiboBio (Guangzhou, China). At 48 h post-transfection, SW480 and HCT116 cells were collected and lysed in lysis buffer containing 20 mM pH 7.5TRIS-HCl, 5 mM MgCl_2_, 100 mM NaCl, and 0.05% Igepal, protease inhibitors, 1 mM DTT, and 60 U/ml Superase-In. The cell lysates were cultured with M-280 streptaviden magnetic beads (Sigma) for 3 h at 4 °C and washed five times by ice-cold lysis buffer. The bound RNAs were subjected to qRT-PCR analysis.

### Mouse xenograft model

The effect of hsa_circ_0137008 on the growth of CRC in vivo was measured by the xenograft experiments. Five-week-old BALB/c nude mice (*N* = 10) were acquired from Vital River Laboratories (Beijing, China) and handled in strict with the procedures approved by the Ethics Committee of The Second Affiliated Hospital of Guangzhou University of Chinese Medicine. SW480 cells stably expressing circ_0137008 were inoculated into nude mice. Tumor size was monitored every 7 days and the volume of xenograft tumors was calculated according to the following formula: Volume = 0.52 × length × width^2^. At the 35th days after inoculation, mice were killed and the tumors were resected, photographed and weighed.

### Statistical analyses

Statistical analysis was performed by the Statistical Program for Social Sciences (SPSS) 20.0 (IBM, NY, USA) software. The data represent the mean ± standard deviation (SD) of at least three independent experiments. The difference between groups was analyzed by using one-way ANOVA or Student’s *t* test. Spearman tests were used to analyze the correlation between hsa_circ_0137008 with miR-338-5p. A *P*-value of less than 0.05 was considered as statistically significant.

## Results

### Hsa_circ_0137008 expression was significantly down-regulated in CRC tissues and cell lines

**Fig. 1:**
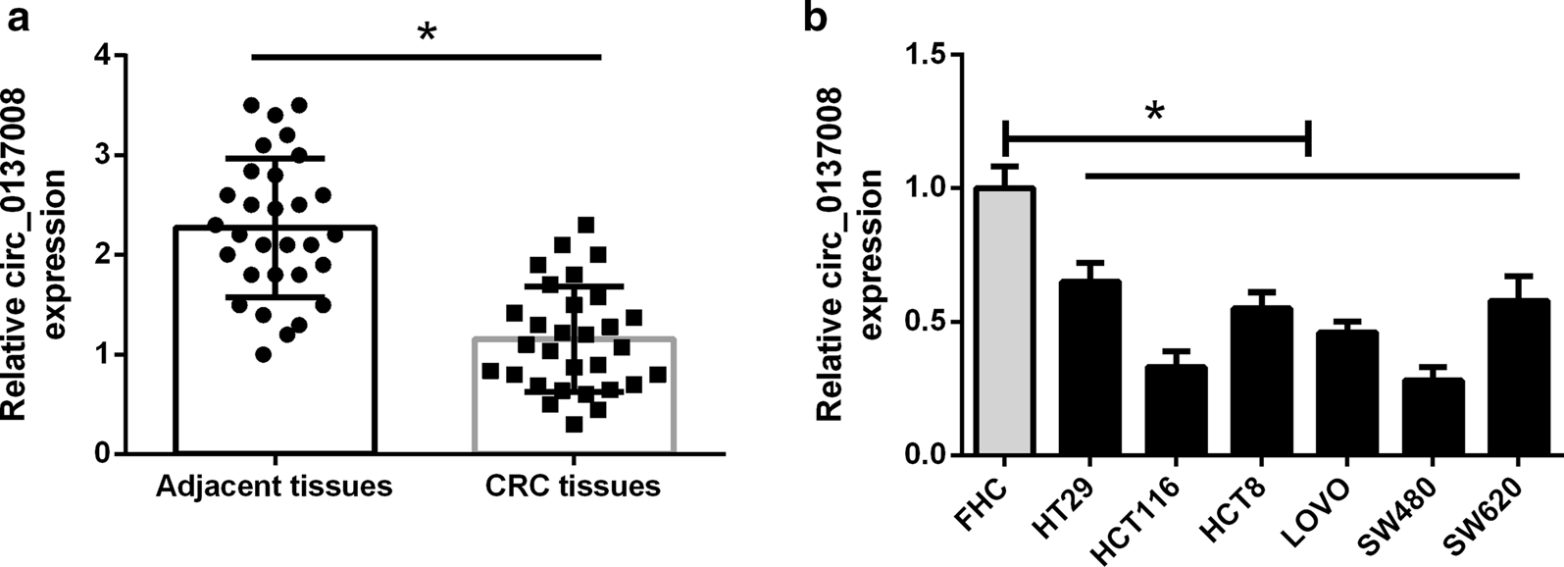
The expression of hsa_circ_0137008 in CRC tissues, adjacent normal tissues, and CRC cell lines. **a** The expression levels of hsa_circ_0137008 in CRC tissues and adjacent normal tissues were detected by qRT-PCR. **b** The expression of hsa_circ_0137008 in FHC cells and CRC cell lines (HT29, HCT116, HCT8, LOVO, SW480, and SW620) was determined by qRT-PCR. *P < 0.05

First, we detected the expression of hsa_circ_0137008 in CRC tissues and adjacent normal tissues, using qRT-PCR. The results showed that hsa_circ_0137008 expression was remarkably down-regulated in CRC tissues compared with that in their adjacent normal tissues (Fig. 1a). Then, we confirmed the differential expression of hsa_circ_0137008 in CRC cell lines and FHC cells. As illustrated in Fig. 1b, the expression of hsa_circ_0137008 in CRC cell lines was much lower than that in FHC cells. Since the expression of hsa_circ_0137008 was greatly lower in SW480 and HCT116 cells than that in the other CRC cells, SW480 and HCT116 cells were selected for the following experiments.

### Overexpression of hsa_circ_0137008 inhibited CRC cell proliferation

**Fig. 2:**
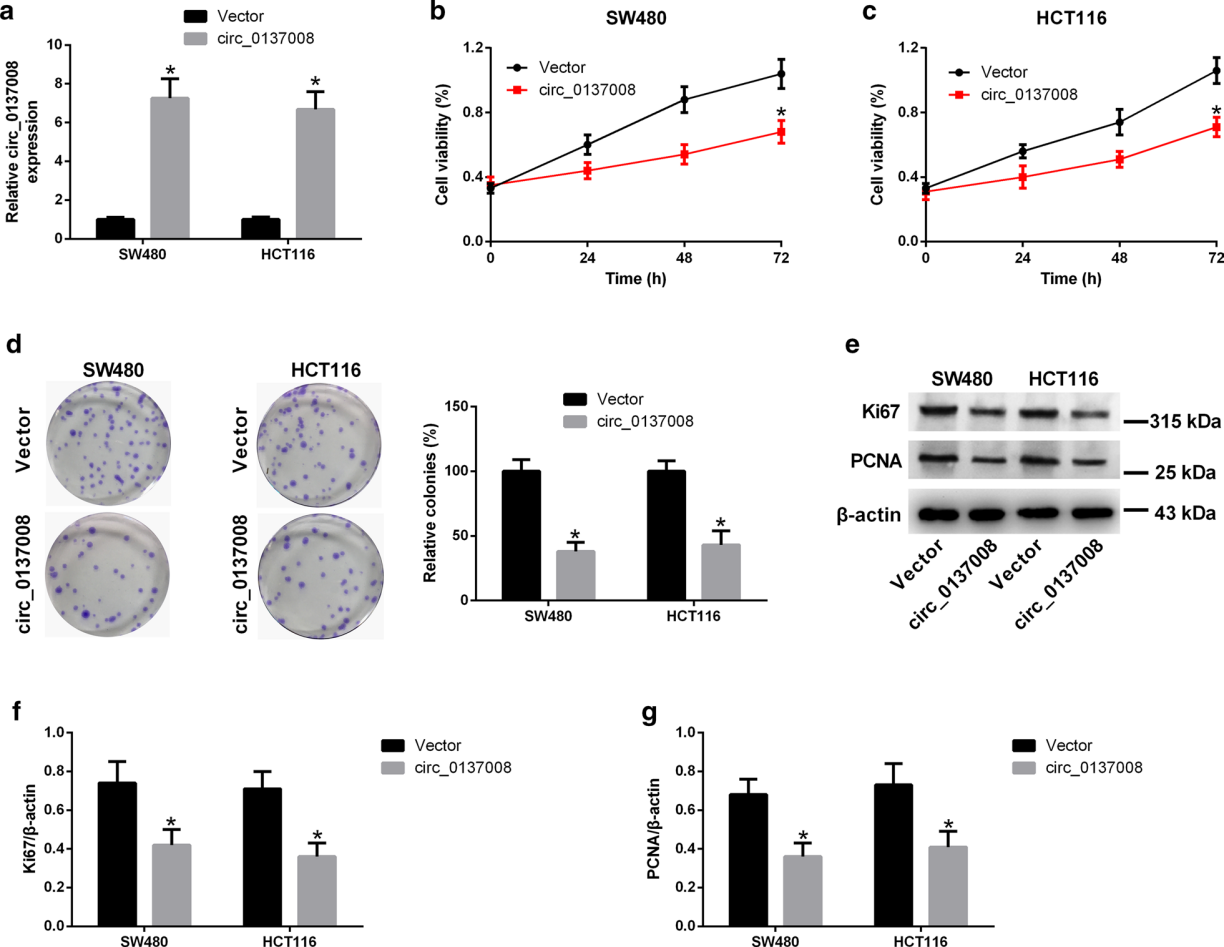
Hsa_circ_0137008 overexpression inhibited the proliferation of CRC cells. SW480 and HCT116 cells were transfected with pcDNA3.1-circ_0137008 or Vector. **a** The expression of hsa_circ_0137008 in SW480 and HCT116 cells were determined following pcDNA3.1-circ_0137008 or Vector transfection. **b**, **c** CCK-8 assay was used to examine the viability of SW480 and HCT116 cells transfected with pcDNA3.1-circ_0137008 or Vector. **d** The colony formation abilities of SW480 and HCT116 cells were evaluated by colony formation assay. **e** The expression of Ki67 and PCNA was determined using western blot. **f**, **g** Results of quantitative analysis of the Ki67 and PCNA protein levels were shown. *P < 0.05

Since hsa_circ_0137008 was downregulated in CRC, we upregulated the expression of hsa_circ_0137008 to assess its biological functions in CRC. qRT-PCR analysis indicated that the expression of hsa_circ_0137008 was evidently increased in SW480 and HCT116 cells transfected with pcDNA3.1-circ_0137008 compared with that in SW480 and HCT116 cells transfected with Vector (Fig. 2a). The results of CCK-8 assay suggested that upregulation of hsa_circ_0137008 decreased the viability of SW480 and HCT116 cells compared with the Vector group (Fig. 2b, c). Similarly, hsa_circ_0137008 overexpression obviously reduced the colony formation ability of SW480 and HCT116 cells in colony formation assay (Fig. 2d). In Fig. 2e, representative images of western blot assays were shown. Furthermore, western blot revealed that upregulation of hsa_circ_0137008 caused a decrease in Ki67 and PCNA expression in SW480 and HCT116 cells (Fig. 2f, g).

### Upregulation of hsa_circ_0137008 inhibited CRC cell migration, invasion, and EMT

**Fig. 3:**
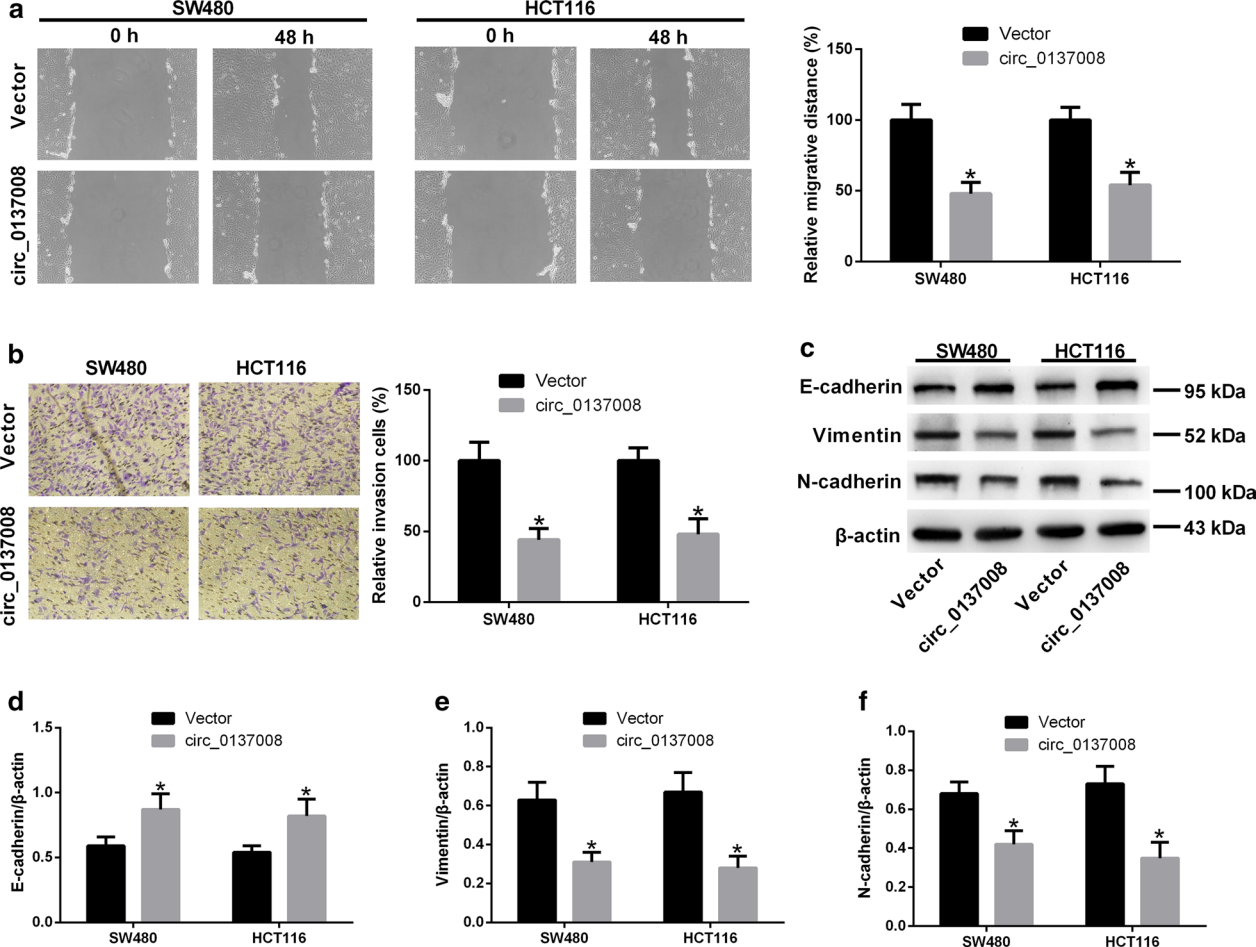
Hsa_circ_0137008 inhibited the migration, invasion, and EMT of CRC cells. SW480 and HCT116 cells were transfected with pcDNA3.1-circ_0137008 or Vector. **a** The migration abilities of SW480 and HCT116 cells was examined using wound healing assay. **b** The invasion abilities of SW480 and HCT116 cells was examined using transwell invasion assay. **c** The expression of E-cadherin, vimentin and N-cadherin was detected using western blot. **d**–**f** Results of quantitative analysis of the E-cadherin, vimentin and N-cadherin protein levels were shown. *P < 0.05

Next, we explored the effect of hsa_circ_0137008 on CRC cell migration and invasion using wound-healing assay and transwell invasion assay. The results showed that upregulation of hsa_circ_0137008 led to a marked decrease in cell migration and invasion in SW480 and HCT116 cells (Fig. 3a, b). To determine the effect of hsa_circ_0137008 on CRC cell EMT, we detected the expression of E-cadherin, vimentin, and N-cadherin in SW480 and HCT116 cells transfected with pcDNA3.1-hsa_circ_0137008 or Vector. Representative images of western blot assays were shown in Fig. 3c. As shown in Fig. 3d–f, the expression of E-cadherin was remarkably increased while the expression of vimentin and N-cadherin was decreased in SW480 and HCT116 cells transfected with pcDNA3.1-hsa_circ_0137008 compared with SW480 and HCT116 cells transfected with Vector.

### Hsa_circ_0137008 served as a sponge for miR-338-5p

**Fig. 4:**
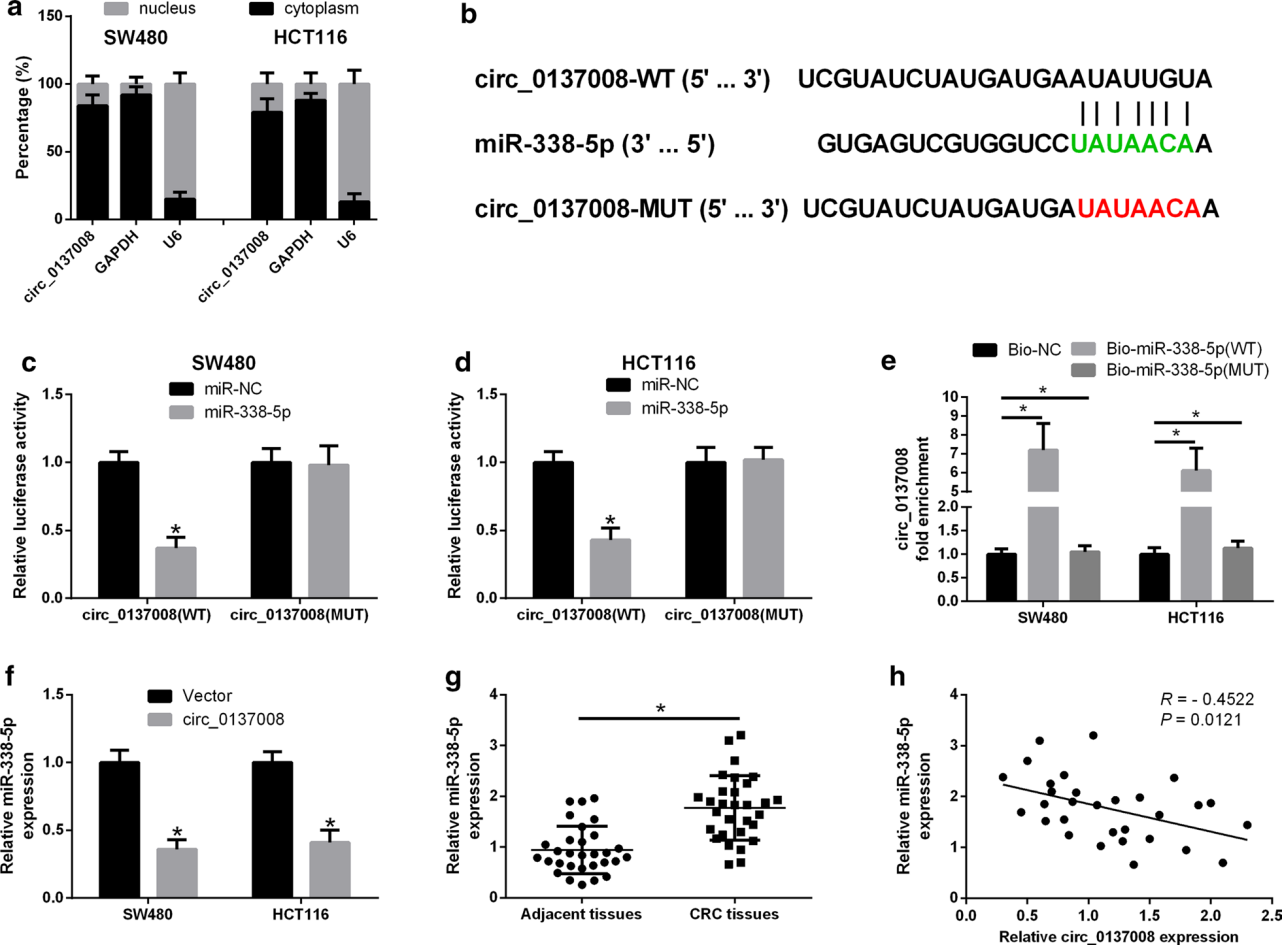
Hsa_circ_0137008 regulated CRC progression by sponging miR-338-5p. **a** The subcellular location assay was performed to determine the cellular location of hsa_circ_0137008 in SW480 and HCT116 cells. **b** The miR-338-5p-binding sites in hsa_circ_0137008 sequence was shown. **c**, **d** Luciferase reporter assay was carried out to determine the interaction between hsa_circ_0137008 and miR-338-5p. **e** RNA pull down assay was utilized to investigate the binding of hsa_circ_0137008 and miR-338-5p. **f** qRT-PCR analysis of miR-338-5p expression in SW480 and HCT116 cells transfected with pcDNA3.1-circ_0137008 or Vector. **g** RT-PCR analysis of mi-338-5p expression in CRC tissues and their adjacent normal tissues. **h** Correlation analysis of hsa_circ_0137008 and miR-338-5p expression in CRC tissues. *P < 0.05

To evaluate the cellular location of hsa_circ_0137008, the nuclear and cytoplasmic RNA was extracted from SW480 and HCT116 cells and then assayed for hsa_circ_0137008 expression using qRT-PCR. We found that hsa_circ_0137008 mainly expressed in the cytoplasm of SW480 and HCT116 cells (Fig. 4a). Subsequently, in order to explore whether hsa_circ_0137008 functions as miRNA sponges in CRC tumorigenesis, we utilized the online software CircInteractome to predict the miRNAs downstream of hsa_circ_0137008. We found that the sequence of hsa_circ_0137008 contains the miR-338-5p-binding sites (Fig. 4b). Then, luciferase reporter assays were used to validate the interaction between hsa_circ_0137008 and miR-338-5. SW480 and HCT116 cells were co-transfected with circ_0137008-WT or circ_0137008-MUT and miR-NC or miR-338-5p. The results suggested that the luciferase activity of reporter containing circ_0137008-WT was remarkably reduced in SW480 and HCT116 cells transfected with miR-338-5p compared with that in cells transfected with miR-NC. However, the luciferase activity of reporter containing circ_0137008-MUT was unaffected in SW480 and HCT116 cells following miR-338-5p or miR-NC transfection (Fig. 4c, d). In line with this, RNA pull-down assays showed that hsa_circ_0137008 was much enriched in the Bio-miR-338-5p (WT) group compared with that in the Bio-miR-NC group, while the enrichment of hsa_circ_0137008 was unaltered in the Bio-miR-338-5p (MUT) group compared with that in the Bio-miR-NC group (Fig. 4e). In addition, we found that the expression of miR-338-5p was markedly decreased in SW480 and HCT116 cells transfected with pcDNA3.1-circ_0137008 (Fig. 4f). Besides, increased expression of miR-338-5p was discovered in CRC tissues compared with that in adjacent normal tissues (Fig. 4g). Spearman’s correlation analysis suggested that miR-338-5p expression was negatively correlated with hsa_circ_0137008 expression in CRC tissues (Fig. 4h).

### Hsa_circ_0137008 regulated CRC cell proliferation by sponging miR-338-5p

**Fig. 5:**
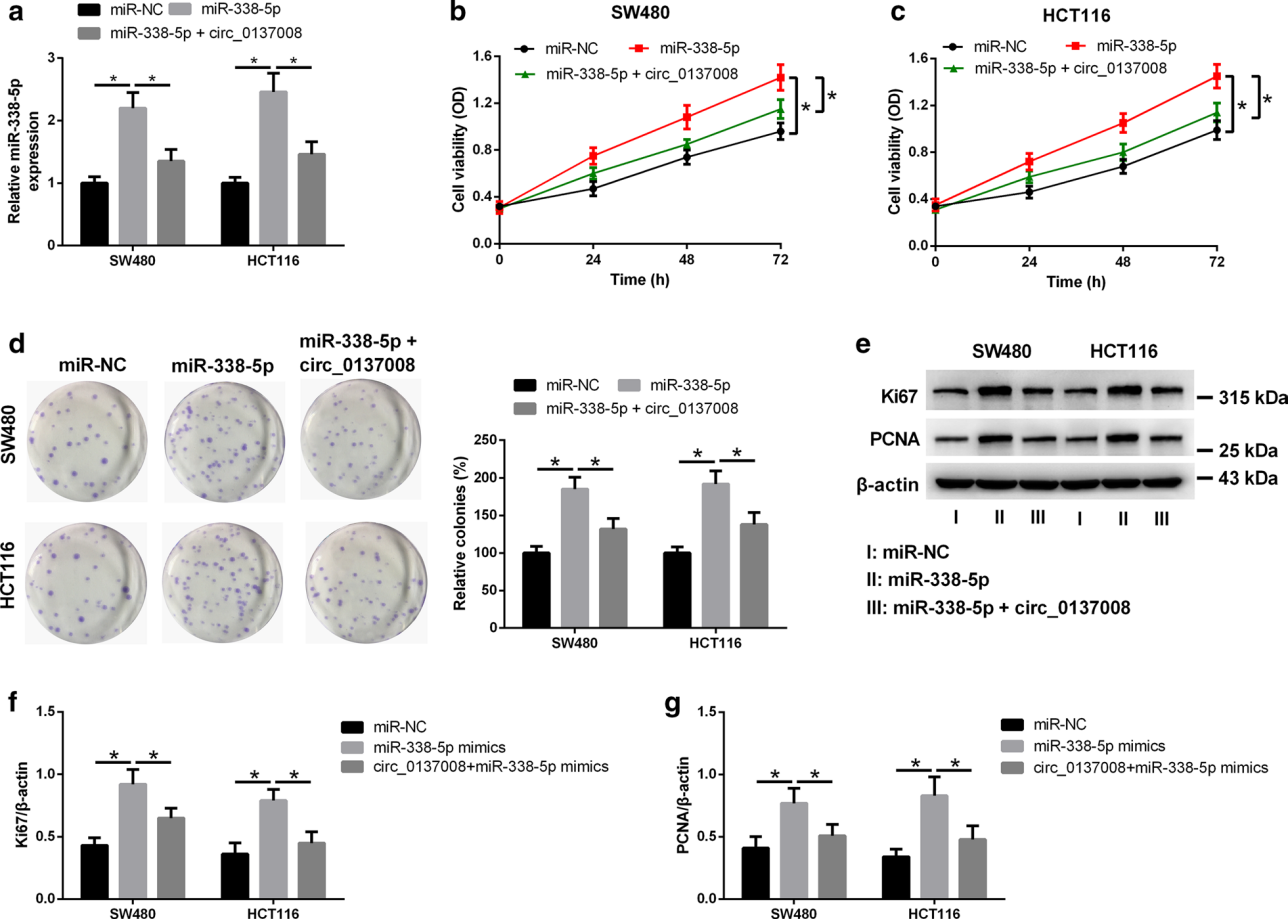
Upregulation of hsa_circ_0137008 attenuated the effect of miR-338-5p on CRC cell proliferation. **a** The expression levels of miR-338-5p were detected in SW480 and HCT116 cells transfected with miR-NC, miR-338-5p mimic or pcDNA3.1-circ_0137008. **b**, **c** The viability of SW480 and HCT116 cells was examined by CCK-8 assay. **d** The colony formation abilities of SW480 and HCT116 cells was determined by colony formation assay. **e** The expression of Ki67 and PCNA was determined using western blot. **f**, **g** Results of quantitative analysis of the Ki67 and PCNA protein levels were shown. *P < 0.05

The above results indicated that hsa_circ_0137008 acted as a sponge of miR-338-5p in CRC cells. We speculated that hsa_circ_0137008 might modulate CRC progression via sponging miR-338-5p. To test this, SW480 and HCT116 cells were transfected with miR-338-5p mimic alone or with pcDNA3.1-circ_0137008. The results of qRT-PCR assays discovered that the expression of miR-338-5p was up-regulated in SW480 and HCT116 cells transfected with miR-338-5p mimic, but co-transfection of SW480 and HCT116 cells with pcDNA3.1-circ_0137008 and miR-338-5p mimic abated this effect (Fig. 5a). Further, upregulation of miR-338-5p enhanced the viability of SW480 and HCT116 cells, which was markedly mitigated following pcDNA3.1-circ_0137008 transfection (Fig. 5b, c). Likewise, colony formation assay suggested that transfection of miR-338-5p mimic increased the colony formation ability of SW480 and HCT116 cells, but this increase was blocked after transfection of pcDNA3.1-circ_0137008 (Fig. 5d). Representative images of western blot assay were presented (Fig. 5e). Moreover, upregulation of miR-338-5p increased the expression of Ki67 and PCNA in SW480 and HCT116 cells. However, upregulation of hsa_circ_0137008 markedly attenuated the miR-338-5p-mediated increase of Ki67 and PCNA expression (Fig. 5f, g).

### Upregulation of hsa_circ_0137008 attenuated the effect of miR-338-5p on CRC cell migration, invasion, and EMT

**Fig. 6:**
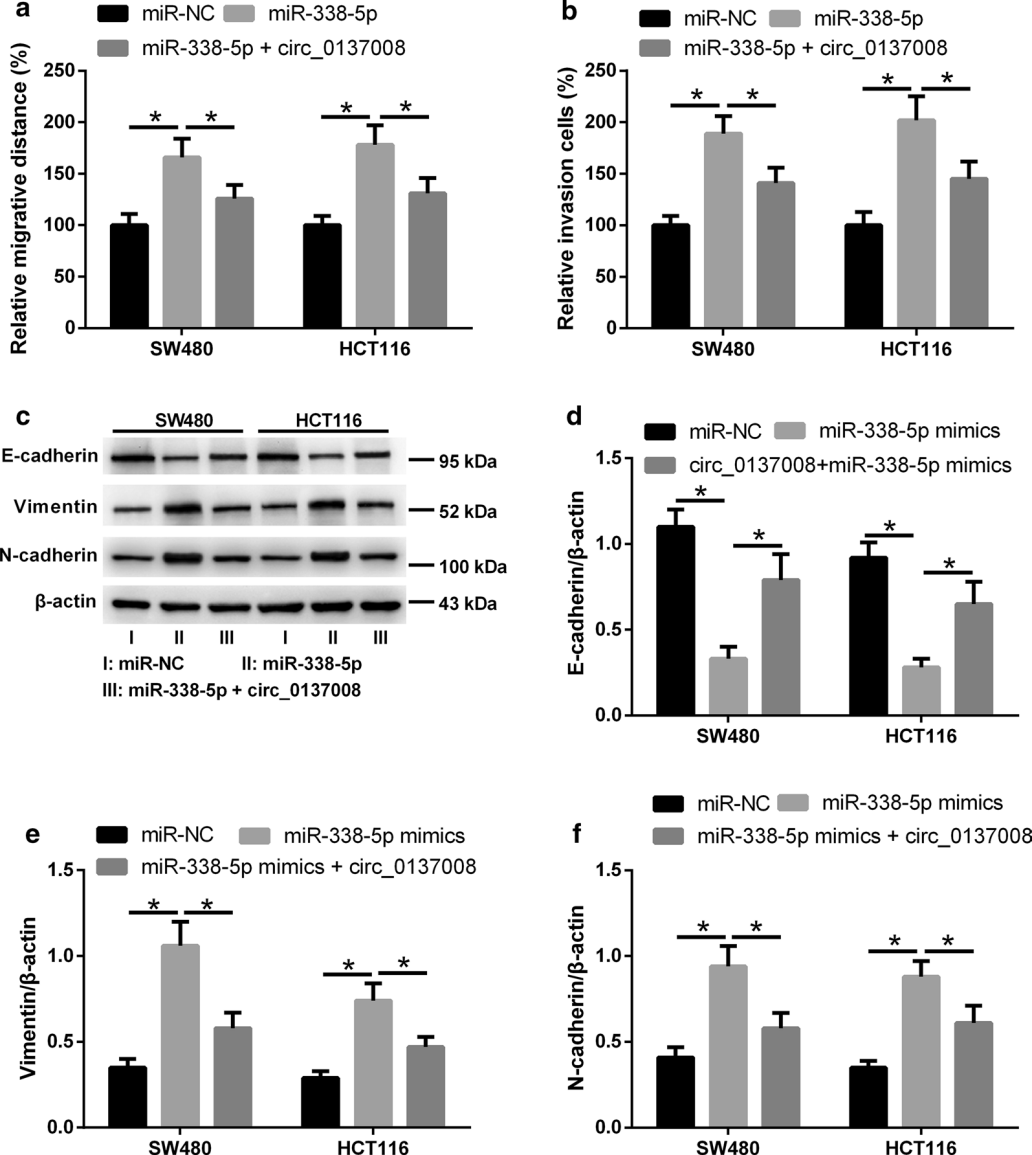
Upregulation of hsa_circ_0137008 attenuated the effect of miR-338-5p on CRC cell migration, invasion, and EMT. SW480 and HCT116 cells were transfected with miR-NC, miR-338-5p mimic alone or with pcDNA3.1-circ_0137008. **a** The migration ability of SW480 and HCT116 cells was examined using wound healing assay. **b** The invasion ability of SW480 and HCT116 cells was examined using transwell invasion assay. **c** The expression of E-cadherin, vimentin and N-cadherin was detected using western blot. **d**–**f** Results of quantitative analysis of the E-cadherin, vimentin and N-cadherin protein levels were shown. *P < 0.05

To explore whether hsa_circ_0137008 regulates CRC cell migration, invasion, and EMT by sponging miR-338-5p, SW480 and HCT116 cells were transfected with miR-338-5p mimic alone or with pcDNA3.1-circ_0137008. Upregulation of miR-338-5p promoted the migration and invasion of SW480 and HCT116 cells, and this action was mitigated following pcDNA3.1-circ_0137008 transfection (Fig. 6a, b). Representative western blot images were shown in Fig. 6c. Additionally, upregulation of miR-338-5p decreased the expression of E-cadherin, but increased the expression of vimentin and N-cadherin in SW480 and HCT116 cells, however, these changes induced by miR-338-5p were obviously reversed by upregulation of hsa_circ_0137008 (Fig. 6d–f).

### Hsa_circ_0137008 upregulation inhibited CRC growth in vivo

**Fig. 7:**
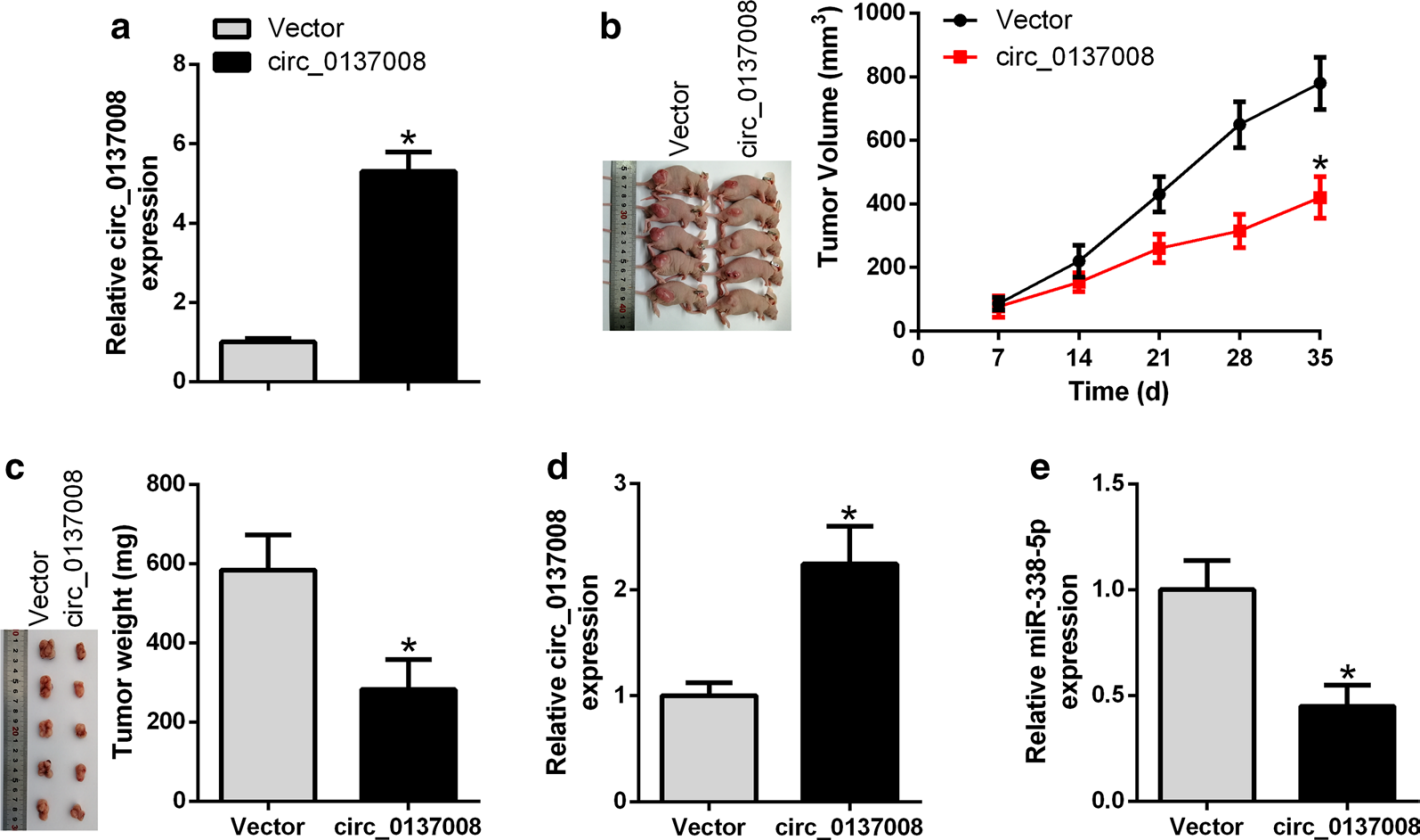
Upregulation of hsa_circ_0137008 inhibited CRC growth in vivo. Nude mice were injected with SW480 cells stably expressing hsa_circ_0137008 and Vector. **a** Stable high-level expression of hsa_circ_0137008 was validated in SW480 cells stably expressing hsa_circ_0137008. **b** Tumor volume was calculated at 7, 14, 21, 28 and 35 days after injection. c Tumor weight was measured at the end of the experiment. **d**, **e** The expression levels of hsa_circ_0137008 and miR-338-5p were detected by qRT-PCR. *P < 0.05

Given the functional role of hsa_circ_0137008 in vitro, we assessed the effect of hsa_circ_0137008 on CRC growth in vivo. The expression level of hsa_circ_0137008 in SW480 cells stably expressing hsa_circ_0137008 was higher than that in SW480 cells stably expressing empty Vector (Fig. 7a). As described in Fig. 7b, c, xenograft tumors from cells expressing hsa_circ_0137008 represented reduced tumor volume and tumor weight relative to tumors from cells expressing Vector. In addition, elevated expression of hsa_circ_0137008 was found in xenograft tumors from cells expressing hsa_circ_0137008 compared with that in tumors from cells expressing Vector (Fig. 7d). Besides, the expression of miR-338-5p was much lower in xenograft tumors from cells expressing hsa_circ_0137008 compared with that in tumors from cells expressing Vector (Fig. 7e).

## Discussion

Recently, circRNAs have been widely studied because of their characteristics, including stable, abundant, conserved, and diverse [24–27]. Deregulation of circRNA has been proposed to be implicated in the development of human cancers, including CRC. For instance, circITGA7 sequestered miR-370-3p away from neurofibromin 1 and then inhibit the activation of Ras signaling, thereby resulting in the suppression of the proliferation and metastasis of CRC cells [28]. circCCDC66 has been reported to be upregulated in CRC and its downregulation repressed the proliferation, migration, invasion and anchorage-independent growth of CRC cells [29]. These literatures implied that the development of CRC is often accompanied by abnormal expression of circRNAs, and circRNAs play a critical role in regulation of various biological processes of cancer cells, which is tightly linked to the initiation and progression of CRC. As a newly discovered circRNA, however, the contribution of hsa_circ_0137008 to CRC carcinogenesis has never been defined. Herein, our findings suggested that the expression levels of hsa_circ_0137008 were remarkably reduced in CRC tissues and cell lines. Overexpression of hsa_circ_0137008 could inhibit the proliferation, migration and invasion, of CRC cells, as evidenced by reduced expression of Ki67, PCNA, vimentin and N-cadherin and elevated expression of E-cadherin. Moreover, upregulation of hsa_circ_0137008 inhibited the growth of CRC in vivo. These results strongly supported the idea that hsa_circ_0137008 functioned as a tumor suppressor in CRC carcinogenesis. These findings suggested that hsa_circ_0137008 may be a potential biomarker for diagnosis and prediction of prognosis of patients with CRC.

Up to now, accumulating evidence suggested that circRNAs exert their biological functions via serving as competing endogenous RNA during the tumorigenesis of human cancers [6, 30, 31]. As an example, overexpression of hsa_circ_0000673 suppressed the proliferation and invasion of gastric cancer cells by sponging miR-532-5p [32]. Moreover, circRNA_LARP4 has been demonstrated to function as a sponge of miR-424 to regulate the progression of gastric cancer [33]. In bladder cancer, circRNA-MYLK served as miR-29a sponge and then activated VEGFA/VEGFR2 and downstream Ras/ERK signaling pathway, thereby promoting the proliferation, metastasis and epithelial-mesenchymal transition of bladder cancer cells [34]. In hepatocellular carcinoma, circADAMTS13 was downregulated in hepatocellular carcinoma tissues and its expression was negatively correlated with tumor size. circADAMTS13 interacted with miR-484 and then suppressed the proliferation of hepatocellular carcinoma cells [35]. These studies demonstrated that circRNAs can act as miRNA “sponges”, thus restraining their ability to target mRNAs. Similar to the above researches, we identified the “sponge” role of hsa_circ_0137008 using luciferase reporter assay and RNA pull-down assay. Functionally, gain-of-function experiments demonstrated that hsa_circ_0137008 the cell proliferation, migration and invasion abilities in two CRC cell lines.

Several researches have proved that miR-338-5p exerts distinct functional roles in different types of cancers. For instance, in esophageal squamous cell carcinoma, miR-338-5p repressed the proliferation, migration, and invasion of CE-81T cells, as well as sensitized CE-81T cells to cisplatin through inhibiting the expression of fermitin family homolog 2, suggesting the anti-oncogenic role of miR-338-5p [36]. In glioma, upregulation of miR-338-5p repressed the proliferation, metastasis and stemness abilities, and promoted the apoptosis and senescence of glioma cells [37]. On the contrary, another study showed that miR-338-5p is associated with tumor staging, distant metastasis and poor prognosis in CRC patients. Upregulation of miR-338-5p enhanced the metastasis of CRC cells through inhibiting phosphatidylinositol 3-kinase, catalytic subunit type 3-mediated autophagy pathway [38]. Consistent with this previous study in CRC, our results of gain-of-function experiments showed that miR-338-5p is upregulated in CRC and functions as an oncogene in vitro. To be specific, upregulation of miR-338-5p promoted the proliferation, migration and invasion of SW480 and HCT116 cells, as indicated by increased expression of Ki67, PCNA, vimentin and N-cadherin and decreased expression of E-cadherin. Importantly, upregulation of hsa_circ_0137008 attenuated the promoting effects of miR-338-5p on CRC cell proliferation, migration and invasion, indicating that hsa_circ_0137008 modulates the progression of CRC by sponging miR-338-5p. Our findings elucidated the upstream regulatory mechanism of ectopic expresson of miR-338-5p in CRC. Beyond miRNA “sponge”, other potential functions of the differentially expressed hsa_circ_0137008 need further investigation in CRC.

## Conclusion

In conclusion, our findings implied that upregulation of hsa_circ_0137008 inhibited the progression of CRC through inhibiton of miR-338-5p. Our study therefore identified a novol regulatory mechanism of tumorigenesis and metastasis in CRC, contributing to a better understanding of the role of circRNAs in CRC progression. Hsa_circ_0137008 may be developed as a prognosis predictor and a promising therapeutic target for CRC.

## Data Availability

The datasets used and/or analyzed during the current study are available from the corresponding author on reasonable request.

## Abbreviations

CCK-8: Cell Counting Kit-8
circRNAs: Circular RNAs
CRC: Colorectal cancer
EMT: Epithelial to mesenchymal transition
FBS: Fetal bovine serum
miR-338-5p: MicroRNA-338-5p
miRNAs: MicroRNAs
RPMI-1640: Roswell Park Memorial Institute-1640 medium
qRT-PCR: Quantitative real-time PCR
